# Positive Treatment Expectations Shape Perceived Medication Efficacy in a Translational Placebo Paradigm for the Gut-Brain Axis

**DOI:** 10.3389/fpsyt.2022.824468

**Published:** 2022-03-24

**Authors:** Sven Benson, Nina Theysohn, Julian Kleine-Borgmann, Laura Rebernik, Adriane Icenhour, Sigrid Elsenbruch

**Affiliations:** ^1^Institute for Medical Education, Center for Translational Neuro- and Behavioral Sciences, University Hospital Essen, University of Duisburg-Essen, Essen, Germany; ^2^Institute of Medical Psychology and Behavioral Immunobiology, Center for Translational Neuro- and Behavioral Sciences, University Hospital Essen, University of Duisburg-Essen, Essen, Germany; ^3^Institute of Diagnostic and Interventional Radiology and Neuroradiology, University Hospital Essen, University of Duisburg-Essen, Essen, Germany; ^4^Department of Neurology, Center for Translational Neuro- and Behavioral Sciences, University Hospital Essen, University of Duisburg-Essen, Essen, Germany; ^5^Department of Medical Psychology and Medical Sociology, Ruhr University Bochum, Bochum, Germany

**Keywords:** treatment expectations, placebo, suggestions, visceral pain, gut-brain axis, patient-reported outcomes, treatment satisfaction, pain perception

## Abstract

Placebo research has established the pivotal role of treatment expectations in shaping symptom experience and patient-reported treatment outcomes. Perceived treatment efficacy constitutes a relevant yet understudied aspect, especially in the context of the gut-brain axis with visceral pain as key symptom. Using a clinically relevant experimental model of visceral pain, we elucidated effects of pre-treatment expectations on post-treatment perceived treatment efficacy as an indicator of treatment satisfaction in a translational placebo intervention. We implemented positive suggestions regarding intravenous treatment with a spasmolytic drug (in reality saline), herein applied in combination with two series of individually calibrated rectal distensions in healthy volunteers. The first series used distension pressures inducing pain (pain phase). In the second series, pressures were surreptitiously reduced, modeling pain relief (pain relief phase). Using visual analog scales (VAS), expected and perceived treatment efficacy were assessed, along with perceived pain intensity. Manipulation checks supported that the induction of positive pre-treatment expectations and the modeling of pain relief were successful. Generalized Linear Models (GLM) were implemented to assess the role of inter-individual variability in positive pre-treatment expectations in perceived treatment efficacy and pain perception. GLM indicated no association between pre-treatment expectations and perceived treatment efficacy or perceived pain for the pain phase. For the relief phase, pre-treatment expectations (*p* = 0.024) as well as efficacy ratings assessed after the preceding pain phase (*p* < 0.001) were significantly associated with treatment efficacy assessed after the relief phase, together explaining 54% of the variance in perceived treatment efficacy. The association between pre-treatment expectations and perceived pain approached significance (*p* = 0.057) in the relief phase. Our data from an experimental translational placebo intervention in visceral pain support that reported post-treatment medication efficacy is shaped by pre-treatment expectations. The observation that individuals with higher positive expectations reported less pain and higher treatment satisfaction after pain relief may provide first evidence that perceived symptom improvement may facilitate treatment satisfaction. The immediate experience of symptoms within a given psychosocial treatment context may dynamically change perceptions about treatment, with implications for treatment satisfaction, compliance and adherence of patients with conditions of the gut-brain axis.

## Introduction

Numerous studies on placebo effects in acute and chronic pain have demonstrated the pivotal role of treatment expectations arising within the psychosocial treatment context (reviewed in Refs. 1, 2). While placebo research in pain has a strong tradition, owing to placebo analgesia as one prominent example of expectancy effects on patient-reported outcomes, the large area of visceral pain has played a comparatively minor role in this translational research field (3). Visceral pain is of high clinical relevance, especially in disorders of gut-brain interactions like the irritable bowel syndrome, but also in inflammatory bowel disease (IBD) and a range of other clinical conditions in gastroenterology, gynecology, urology, and psychosomatic medicine (4, 5). Since the notable clinical work by Kaptchuk and colleagues demonstrating the therapeutic potential of placebo interventions in patients with IBS (6, 7), clinical research on visceral pain modulation and impact of expectancy effects on treatment responses in clinical trials in the gastrointestinal field continues to thrive (3, 8–11). This calls for laboratory studies dedicated to elucidating the psychological and neurobiological mechanisms in clinically relevant models of visceral pain.

While existing evidence impressively underscores expectancy effects on visceral pain perception both in healthy volunteers and in clinical conditions involving the gut-brain axis (3, 12), there exist gaps in knowledge that even novel research approaches have not fully captured thus far (13). It is important to understand if and how treatment expectations shape perceived treatment efficacy as a key patient-reported outcome and indicator of overall treatment satisfaction. Indeed, the subjective evaluation of how well a treatment worked is a crucial component of patients’ perspective on quality of healthcare in clinical trials and practice (14, 15). This is increasingly appreciated in placebo research accomplished in patients with somatic pain conditions (16), but remains insufficiently considered in clinical and laboratory studies on underlying psychological mechanisms, especially in the context of visceral pain. In experimental visceral pain, we previously showed that perceived treatment group allocation constitutes an important aspect in symptom reports (17). Specifically, healthy volunteers who believed that they received a potent analgesic drug reported less discomfort induced by rectal distensions and reduced neural activation of several relevant brain regions, including the insula and cingulate cortex, when compared to volunteers who believed that they had received an inert treatment. Further, perceived treatment allocation was impacted by symptom burden in response to experimentally induced acute inflammation (18). These initial findings from experimental studies suggest that expectations and visceral symptom experience shape cognitive processes underlying patients’ evaluations and possibly judgments regarding treatment. Since perceived efficacy of an analgesic treatment is essential to treatment satisfaction and adherence, it is important to model the impact of treatment expectations together with the immediate experience of changes in symptom intensity in experimental placebo research.

In a translational placebo intervention for visceral pain, we elucidated whether and to what extent interindividual variability in positive treatment expectations arising from positive treatment information within a standardized treatment context is associated with perceived treatment efficacy and visceral pain perception. To induce positive treatment expectations in healthy volunteers, we capitalized on an established placebo intervention which consists of positive suggestions regarding treatment with an intravenous spasmolytic drug (in reality saline) (19–23). Repeated rectal distensions, carried out following placebo administration, were individually calibrated to be initially painful, and subsequently surreptitiously lowered in intensity to model pain relief. This approach was inspired by the clinical treatment reality of patients experiencing fluctuating symptoms and/or delayed treatment onset, which is highly relevant in conditions of acute or chronic visceral pain where clear and immediate treatment success may be particularly difficult to achieve. Initial analyses were carried out to verify the successful implementation of distinct perceptual experiences by different distension pressures (manipulation check), as well as to ascertain the effective induction of positive treatment expectations in the placebo intervention group when compared to a reference group (treatment check). Primary analyses were computed within all positively instructed individuals (placebo group) with generalized linear models (GLMs) for the pain and pain relief phases, respectively, using treatment efficacy and perceived intensity ratings as response variables.

## Materials and Methods

### Participants

Healthy participants were recruited by public advertisement seeking volunteers for an experimental study designed to test psychological mechanisms underlying effects of different drugs on experimentally induced visceral symptoms including pain. For the purposes of this report, we analyzed selected behavioral measures from a dataset of a total of *N* = 60 healthy participants who were at inclusion randomized (with a 2:1 randomization) to undergo an established placebo intervention consisting of positive drug-related treatment suggestions (*N* = 40, placebo group) or to receive no drug-related suggestions (*N* = 20, reference group) prior to experiencing phasic visceral stimuli (details below). All volunteers were recruited *de novo* for this study and had to be naïve with respect to both the distension model as well as to any prior experimental placebo or nocebo study carried out by our research group. The recruitment and in-depth screening procedures consisted of an initial semi-structured telephone screening (conducted by author LR), followed by a structured personal interview and a brief general medical examination, including a digital rectal exam (conducted by study physician, author NT). Exclusion criteria included a body mass index (BMI) <18 or >30, age <18 or >45 years, any known medical or psychological/psychiatric clinical conditions, and any current medication use (except occasional use of non-prescription over-the-counter drugs for minor allergies, benign headaches). Participants were also screened for self-reported substance abuse, including number of alcoholic drinks/week (>4/week led to exclusion), smoking (>10 cigarettes per week led to exclusion), and use of other recreational drugs (any reported use within past 3 months led to exclusion). Current anxiety or depression symptoms above the published cut-off values (i.e., scores ≥ 8) on the Hospital Anxiety and Depression Scale (HADS) (24), and frequent gastrointestinal (GI) symptoms within past 3 months suggestive of an undiagnosed GI condition based on self-reports during phone screening or on a gastrointestinal symptom questionnaire (items assessed: diarrhea, constipation, vomiting, nausea, lower abdominal pain, upper abdominal pain, heartburn, post-prandial fullness, bloating, loss of appetite) (25) completed during the personal interview also led to exclusion from participation. Peri-anal tissue damage (e.g., painful hemorrhoids or fissures which may interfere with balloon placement) upon digital examination during the physical examination were also an exclusion criterium. Since the study was originally accomplished to elucidate pain-related brain mechanisms (data not reported herein), the usual MR-specific exclusion criteria also applied (i.e., claustrophobia, pregnancy or ferromagnetic implants, and any evidence of structural brain abnormalities, verified by a neuroradiologist, author NT). Pregnancy was ruled out using a commercially available pregnancy test on the study day (Biorepair GmbH, Sinsheim, Germany, sensitivity 10 mIU/ml). In addition to HADS, we also herein report trait anxiety assessed with the trait version of the Spielberger State-Trait-Anxiety Inventory (STAI-T) (26). Ethics approval was granted by the University Hospital Essen Ethics Committee (permit no. 08-3823). All volunteers gave written informed consent, and were paid for their participation.

### Experimental Procedures

**FIGURE 1:**
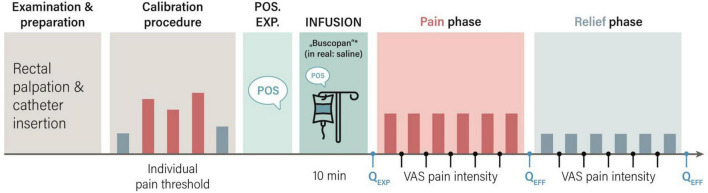
Study procedures for the placebo group. After rectal catheter placement, individual pain thresholds were determined in a calibration procedure. The study physician verbally delivered treatment suggestions about treatment with a spasmolytic drug (in reality saline) in order to induce positive treatment expectations (Pos. Exp.), and the intravenous infusion was started. In the subsequent pain phase, individual distension pressures inducing pain were applied. In the pain relief phase, pressures were surreptitiously reduced to model pain relief. Using visual analog scales (VAS), expected (*Q*_*EXP*_), and perceived (*Q*_*EFF*_) treatment efficacy were assessed. Perceived distension intensity was assessed after each stimulus, and averaged for the pain and pain relief phases, respectively. *Buscopan: scopolamine butylbromide.

Experiments were conducted within a medical research setting within a clinical MR suite of the University Hospital Essen, Germany. On the study day (see Figure 1 for a time line), a catheter was placed to apply pressure-controlled rectal distensions as a clinically relevant and reliable experimental model for the study of acute visceral pain in healthy individuals as well as in patients with chronic visceral pain (27). Given high interindividual variability in rectal sensitivity in healthy participants (28, 29) and our paradigm requiring precisely titrated pressures for the induction of distinct perceptual intensities in the pain and pain relief phases, respectively, a thresholding procedure was accomplished initially. Individual rectal sensory and pain thresholds were determined using a barostat system (modified ISOBAR 3 device, G & J Electronics, ON, Canada) in accordance with our prior work and recommendations within neurogastroenterology (e.g., 28, 29). Specifically, we utilized a double-random staircase procedure with a series of phasic distensions (duration each 30 s) with random pressure increments of 2–8 mmHg. Pauses of complete balloon deflation (i.e., 0 mmHg pressure) in-between each distension were 30 s. The maximal distension pressure was set at 50 mmHg. For each distension, participants rated the perception on a Likert scale labeled 1 = no perception, 2 = doubtful perception, 3 = sure perception, 4 = little discomfort, 5 = severe discomfort, still tolerable distension and 6 = pain, not tolerable distension. Sensory threshold was defined as pressure when ratings changed from 2 to 3; pain threshold was defined as pressure when ratings changed from 5 to 6. The duration of the thresholding procedure takes approximately 20–30 min, depending on the individual pain threshold.

Based on results of thresholding, individualized pressures aiming to create two distinct stimulation intensities for subsequent application during two series of cued phasic rectal distensions were identified. In the first series, six painful visceral stimuli using distension pressures just below the individual pain threshold were implemented (pain phase). The subsequent second series consisted of six distensions at surreptitiously reduced pressures corresponding to just above the sensory threshold (relief phase). All distensions were cued by a visual signal, and the duration of each distension was 18 s. Pauses of complete balloon deflation in-between distensions were 18 s. Visual analog scale (VAS) were accomplished prior to and after each phase for expectation and efficacy ratings, and after each distension for perceived intensity (details below).

### Induction of Positive Treatment Expectations (Placebo Intervention)

We implemented a previously established translational placebo intervention for the visceral pain modality to induce positive treatment expectations by suggestions, which we have previously applied in studies with healthy volunteers and patients with IBS and IBD (19–23). It essentially builds on deceptive treatment suggestions regarding the intravenous administration of a potent spasmolytic drug with analgesic properties, which is in reality always saline. As previously established (28, 29), positive treatment suggestions included both written and standardized verbal information regarding the intravenous (i.v.) administration of a spasmolytic drug (Butylscopolaminiumbromid) with analgesic properties. Specifically, during recruitment and as part of informed consent, all *N* = 40 participants in the placebo group received positive information verbally and in writing, including detailed drug-related information. On the study day, before the i.v. drip was started, pertinent aspects of the positive instructions, were repeated verbally (“medication reminder communication”) by the study physician (author NT), especially focusing on the pain-relieving properties of the drug (see Figure 1). In line with our previous clinically oriented work (23), the duration of this was recorded as a global measure of patient-provider-communication quantity. The study physician then prepared a syringe clearly labeled with the drug name in full view of participants, and injected its content (in reality saline) into an infusion bottle with saline. Note that as part of the study, we piloted two versions of this positive reminder communication: While all *N* = 40 participants were reminded of the pertinent drug information, half of the sample (*N* = 20) received more detailed and personalized information (i.e., augmented vs. limited medication reminder).

As a reference group, herein used to confirm that the placebo intervention successfully induced positive treatment expectations, *N* = 20 individuals were truthfully informed about administration of an inert substance. Specifically, this reference group was informed about i.v. administration of saline, and written and verbal instructions contained a specific mention of absence of active drug and lack of saline effects. The infusion bottle was clearly marked as “sodium chloride,” and no injection into this bottle was accomplished. Any verbal communication between physician and participant during the i.v. preparation was kept as neutral and “technical” as possible, making no references to treatment or pain.

### Measures

Visual analog scale (VAS, 0–100 mm) ratings were accomplished using an automated response system (LUMItouch™, Photon Control Inc., Burnaby, BC, Canada). Specifically, expected medication efficacy, i.e., positive treatment expectation, was rated on VAS (ends labeled “not at all effective–highly effective”) immediately after the verbal induction of positive treatment expectations by the study physician. After each distension series (i.e., pain and pain relief phases, respectively), perceived treatment efficacy was assessed. Note that all treatment efficacy VAS were specifically phrased to address the expected or the experienced ability of the drug to successfully relieve visceral pain (“How effectively will/did the drug relieve your pain,” ends labeled “not at all–very much”). In addition, each distension was rated on VAS for intensity, and ratings were averaged within each phase for analyses. VAS state tension ratings were accomplished along with efficacy ratings (see Figure 1).

### Statistical Analyses

For initial manipulation/treatment checks, placebo and reference groups were compared with respect to objective and subjective distension-related measures as well as sociodemographic and psychological measures using independent sample *t*-tests and Chi^2^-test. Change in subjective distension perception was calculated with repeated measures ANOVA with the between subject factor group (placebo, reference) and time (pain, relief). Next, to exclude differences between positively instructed participants who received augmented vs. limited medication reminder communication, distension-related outcomes were compared with independent-samples *t*-tests. Data were reported as mean ± standard deviation (SD), effect sizes as Cohen’s d. In case that Levene test for homogeneity of variances was significant, we show corrected df. Data were analyzed with SPSS version 27.0 (IBM Corporation, Armonk, NY, United States).

To address main research aims, i.e., to assess the role of positive treatment expectations, analyses within all positively instructed participants (placebo group, *N* = 40) were performed with RStudio (RStudio Team, Version 1.4.1717, RStudio, PBC, Boston, MA, United States).^1^ Separate generalized linear models (GLMs) were calculated using pre-treatment expectation ratings as exploratory and (a) treatment efficacy for the pain and pain relief phases and (b) perceived distension intensity as response variables. All outcome models were additionally corrected for the following covariates: Tension (VAS), duration of the informed consent procedure (min), stimulus intensity (mmHg), treatment efficacy (VAS, for models addressing pain intensity), pain intensity (VAS, for models addressing treatment efficacy). In supplemental analyses, models were re-computed after exclusion of outliers (in expectation and efficacy ratings, *N* = 5 exclusions), defined outliers as values 2 SD below or above mean. Statistical testing was performed at alpha < 0.05.

## Results

### Participants and Manipulation and Treatment Checks

Consistent with stringent exclusion criteria, healthy male and female participants were overall young and of normal weight, and characterized by low anxiety and depression symptom scores, normal trait anxiety scores, and low gastrointestinal complaints. Mean rectal sensory and pain thresholds were comparable with our previous findings on visceral pain sensitivity in young healthy participants (e.g., 29). No differences in any of these variables except for a small, statistically significant different in HADS depression scores were found between the placebo (*N* = 40) and the reference (*N* = 20) groups (Table 1).

**TABLE 1:** Sample characteristics.

	Placebo (*N* = 40)	Reference (*N* = 20)	*P*
Age (years)	25.9 ± 5.2	24.6 ± 3.0	0.29
Sex (*N* female/*N* male)	20/20	9/11	0.72
Body mass index (kg/m^2^)	23.0 ± 2.8	22.4 ± 2.2	0.34
HADS anxiety score	3.6 ± 2.8	2.6 ± 2.1	0.16
HADS depression score	2.1 ± 2.1	1.0 ± 1.4	0.04
STAI trait score	35.4 ± 7.3	33.1 ± 7.5	0.26
GI symptom score	4.1 ± 2.8	3.6 ± 2.9	0.57
Rectal sensory threshold, mmHg	14.9 ± 3.8	14.2 ± 3.0	0.47
Rectal pain threshold, mmHg	35.0 ± 10.7	34.6 ± 7.4	0.88

*Data are shown as mean ± standard deviation, unless otherwise indicated. No significant group differences were observed between the Placebo and Reference groups (P values indicate results of independent sample t-tests or Chi^2^ tests for sex). GI, gastrointestinal; HADS, Hospital Anxiety and Depression Scale; STAI, State-Trait Anxiety Inventory.*

As manipulation check, we initially ascertained differences between experimental phases with respect to objective and subjective distension-related measures. As intended, distension pressures applied within the pain phase were markedly higher than pressures applied within the pain relief phase, consistent with their selection based on individual thresholds (Supplementary Table 1). Further, the applied distension pressures consistently led to distinct perceptual intensities, i.e., greater perceived intensity during the pain phase and lower perceived intensity during the subsequent pain relief phase (Supplementary Table 1), together supporting the efficacy of experimental manipulations.

Subsequently, the overall efficacy of the placebo intervention was tested by comparing positive expectations in the placebo and reference groups (treatment check). The placebo intervention successfully induced positive treatment expectations, as evidenced by overall significantly higher positive treatment expectations in the placebo group (*N* = 40) when compared to the reference group who received no positive drug-related suggestions [VAS pre-treatment expectations: 69.9 ± 11.8 mm vs. 14.8 ± 22.8 mm; *t*_(40_._1)_ = 10.1, *p* < 0.001, *d* = 3.4].

### Treatment Expectations and Perceived Treatment Efficacy in the Placebo Group

Our primary aim was to assess the role of inter-individual variability in positive pre-treatment expectations in perceived treatment efficacy and symptom perception, which is why our strategy capitalized on variability in the whole sample of all positively instructed participants (placebo group, *N* = 40). To this end, the entire placebo group was analyzed using GLM, irrespective of two slightly different medication reminder communication strategies implemented just prior to placebo administration by the study physician. For the sake of completeness, we provide comparisons of outcome measures for these subgroups (augmented vs. limited, Supplementary Table 2). Briefly, no subgroup differences in outcome measures were observed, but the reminder communication was significantly longer in the augmented subgroup [10.9 ± 2.4 vs. 6.8 ± 1.5 min, *t*_(37)_ = 6.4, *p* < 0.001], which we considered as a covariate in GLM analyses. Further, positive treatment expectations were higher in the augmented subgroup [75.2 ± 9.7 vs. 64.7 ± 11.4 mm on VAS, *t*_(37)_ = 3.1, *p* = 0.003], providing us with variability for primary analyses using GLM.

**FIGURE 2:**
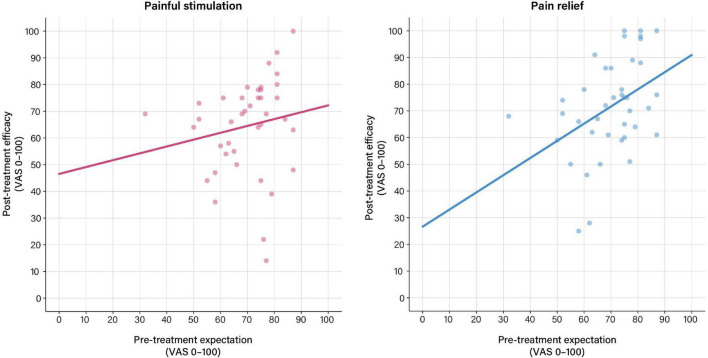
Associations between pre-treatment expectation and perceived treatment efficacy based on general linear models (GLM) calculated using pre-treatment expectation as exploratory and treatment efficacy ratings as response variables. **(Left)** No significant association between pre-treatment expectation and perceived treatment efficacy was found after painful stimulation in the pain phase. **(Right)** For the relief phase, pre-treatment expectations were significantly associated with perceived treatment efficacy (*b* = 0.35, *t* = 2.37, *p* = 0.024). For details, see Table 2. For results after exclusion of outliers, see Supplementary Table 3 and Supplementary Figure 1.

For the pain phase, GLM indicated that pre-treatment expectations were not associated with treatment efficacy assessed after the pain phase (Table 2 and Figure 2). For the relief phase, pre-treatment expectations were significantly associated with treatment efficacy assessed after the relief phase (Table 2 and Figure 2). In this model, pre-treatment expectation (*p* = 0.024) together with efficacy ratings assessed after the preceding pain phase (*p* < 0.001) explained 54% of the variance in perceived treatment efficacy (Table 2).

**TABLE 2:** Predictors of treatment efficacy after pain and relief phases (Results of generalized linear models, GLM).

Treatment efficacy after pain phase (*R*^2^ = 0.17)							
**Predictors**	**Estimates**	**Std. Beta**	**CI**	**Std. CI**	**t**	**p**	**df**
(Intercept)	69.76	–0.00	23.6–116.0	–0.31–0.31	2.96	**0.006**	33
Pre-treatment expectation (VAS)	0.25	0.17	–0.30–0.80	–0.20–0.53	0.89	0.38	33
Perceived intensity for pain phase (VAS)	–0.13	–0.13	–0.45–0.20	–0.48–0.21	–0.76	0.45	33
Stimulus intensity for pain phase (mmHg)	–0.37	–0.21	–1.01–0.27	–0.58–0.16	–1.13	0.27	33
Tension after pain phase (VAS)	0.11	0.18	–0.10–0.32	–0.16–0.52	1.05	0.30	33
Duration of medication reminder communication (minutes)	–0.85	–0.14	–3.03–1.32	–0.49–0.21	–0.77	0.45	33

**Treatment efficacy after relief phase (*R*^2^ = 0.54)**							

**Predictors**	**Estimates**	**Std. Beta**	**CI**	**Std. CI**	**t**	**p**	**df**

(Intercept)	–6.06	–0.00	–46.5–34.4	–0.23–0.23	–0.29	0.77	32
Pre-treatment expectation (VAS)	0.53	0.35	0.09–0.97	0.06–0.64	2.37	**0.024**	32
Treatment efficacy rating for pain phase (VAS)	0.59	0.59	0.33–0.86	0.33–0.86	4.35	**<0.001**	32
Perceived intensity for relief phase (VAS)	0.06	0.06	–0.27–0.39	–0.28–0.40	0.35	0.73	32
Stimulus intensity for relief phase (mmHg)	0.17	0.10	–0.35–0.68	–0.20–0.39	0.64	0.53	32
Tension after relief phase (VAS)	–0.10	–0.15	-0.31–0.12	–0.49–0.19	–0.88	0.39	32
Duration of medication reminder communication (minutes)	–0.06	–0.01	–1.84–1.71	–0.30–0.28	–0.07	0.94	32

*Separate generalized linear models (GLMs) with pre-treatment expectation as exploratory and treatment efficacy ratings as response variables were calculated for the pain and pain relief phases, respectively, in all positively instructed volunteers (N = 40, placebo group). CI, confidence interval; df, degree of freedom; Std., Standardized; t, t value; VAS, visual analog scale. Significant p-values are printed in bold.*

After exclusion of outliers, treatment expectation was significantly associated with treatment efficacy ratings for the pain phase. For the pain relief phase, predictors in the GLM model remained unchanged, however, with a lowered level of significance for pre-treatment expectation (*p* = 0.05) (Supplementary Table 3 and Supplementary Figure 1).

### Treatment Expectations and Perceived Distension Intensity in the Placebo Group

**FIGURE 3:**
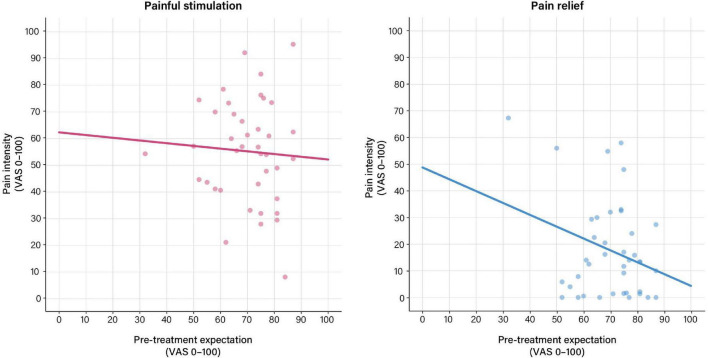
Associations between pre-treatment expectation and perceived distension intensity based on general linear models (GLM) calculated using pre-treatment expectation as exploratory and perceived intensity ratings as response variables. **(Left)** For the pain phase, no significant association between pre-treatment expectation and perceived distension intensity was observed. **(Right)** For the relief phase, the association between pre-treatment expectation and perceived distension intensity approached significance (*b* = 0.30, *t* = –1.97, *p* = 0.057). For details, see Table 3. For results after exclusion of outliers, see Supplementary Table 4 and Supplementary Figure 2.

For the pain phase, perceived pain was only associated with objective stimulus intensity (*p* = 0.028), but not with treatment expectation (Table 3 and Figure 3). For the relief phase, the association between treatment expectation and perceived pain approached significance (*p* = 0.057). In this model, objective stimulus intensity (*p* = 0.008) and tension after the relief phase (*p* = 0.001) were significant covariates, with the model explaining 52% of the variance in perceived distension intensity (Table 3 and Figure 3).

**TABLE 3:** Predictors of pain intensity during pain and relief phases (Results of generalized linear models, GLM).

Subjective pain intensity during pain phase (*R*^2^ = 0.21)							
**Predictors**	**Estimates**	**Std. Beta**	**CI**	**Std. CI**	**t**	**p**	**df**
(Intercept)	23.59	0.00	-29.53–76.71	–0.30–0.30	0.87	0.39	33
Pre-treatment expectation (VAS)	0.16	0.10	–0.41–0.74	–0.26–0.46	0.56	0.58	33
Treatment efficacy rating for pain phase (VAS)	–0.014	–0.13	–0.49–0.21	–0.46–0.20	–0.76	0.45	33
Stimulus intensity for pain phase (mmHg)	0.74	0.40	0.11–1.37	0.06–0.74	2.30	**0.028**	33
Tension after pain phase (VAS)	0.20	0.30	–0.01–0.40	–0.02–0.62	1.86	0.072	33
Duration of medication reminder communication (minutes)	–0.28	–0.04	–2.54–1.98	-0.39–0.30	–0.24	0.81	33

**Subjective pain intensity during relief phase (*R*^2^ = 0.52)**							

**Predictors**	**Estimates**	**Std. Beta**	**CI**	**Std. CI**	**t**	**p**	**df**

(Intercept)	49.24	–0.00	12.49–85.98	–0.24–0.24	2.63	**0.013**	32
Pre-treatment expectation (VAS)	–0.47	0.30	–0.94 – –0.00	–0.61 – –0.00	–1.97	0.057	32
Treatment efficacy rating for relief phase (VAS)	0.19	0.18	–0.09–0.47	–0.09–0.45	1.31	0.20	32
Perceived intensity for pain phase (VAS)	0.19	0.20	–0.10–0.48	–0.10–0.50	1.30	0.20	32
Stimulus intensity for relief phase (mmHg)	–0.71	–0.40	–1.20 – –0.22	–0.67 – –0.12	–2.85	**0.008**	32
Tension after relief phase (VAS)	0.36	0.56	0.16–0.57	0.25–0.88	3.48	**0.001**	32
Duration of medication reminder communication (minutes)	–1.49	–0.24	–3.28–0.29	–0.52–0.05	–1.64	0.11	32

*Separate generalized linear models (GLMs) with pre-treatment expectation as exploratory and perceived intensity ratings as response variables were calculated for the pain and pain relief phases, respectively, in all positively instructed volunteers (N = 40, placebo group). CI, confidence interval; df, degree of freedom; Std., Standardized; t, t value; VAS, visual analog scale. Significant p-values are printed in bold.*

After exclusion of outliers, associations with treatment expectation for the pain or pain relief phases remained non-significant (Supplementary Table 4 and Supplementary Figure 2).

## Discussion

Research into placebo effects has established the pivotal role of treatment expectations in symptom experience, including the experience of acute visceral pain and other burdening symptoms of the gut-brain axis (3). As a crucial component of overall treatment satisfaction, perceived treatment efficacy constitutes a relevant yet understudied element of expectancy effects on patient-reported outcomes, which has thus far not been addressed in visceral pain. In a translational placebo intervention for acute visceral pain, we focused our analyses on interindividual variability in levels of positive expectations in a placebo group, and elucidated perceived treatment efficacy and perceived stimulus intensity after an initial treatment phase modeling pain and a subsequent treatment phase modeling pain relief. To induce positive treatment expectations in naïve healthy participants, we implemented positive drug-related suggestions, i.e., written and verbal information regarding the i.v. administration of a potent spasmolytic drug with analgesic properties. In line with our earlier findings in this placebo intervention (28, 29), positive suggestions successfully induced overall high levels of positive treatment expectations, as evidenced by comparison with expectations in a reference group that had received information regarding saline as an inert substance. The extent of positive expectations within the placebo group was greatest in a subgroup with an optimized (augmented) medication reminder communication accomplished in the immediate treatment context, which we piloted within this project. While not our primary focus herein, this interesting finding expands on work dedicated to the crucial role of patient-provider communication in shaping expectancy effects (30, 31), enhances the generalizability and translation or our experimental work to clinical settings where quantity and quality of communication obviously vary greatly, and effectively provided us with sufficient variability in levels of positive expectations for our primary analyses using general linear models (GLM) in all positively instructed individuals, i.e., the entire placebo group.

Generalized linear models supported that positive pre-treatment expectations were associated with greater perceived treatment efficacy. Effects were greater and more robust to outliers for the pain relief phase, consisting of rectal distensions with surreptitiously reduced pressures, effectively creating the experience of pain relief. In other words, the magnitude of positive treatment expectations scaled with the perception of a more potent analgesic drug after the experience of improved pain. Pre-treatment expectations explained 54% (62% after exclusion of outliers) of the variance in perceived treatment efficacy rated after the pain relief phase, in a model that considered a number of other variables as covariates. Besides treatment expectations, perceived treatment efficacy after the preceding pain phase emerged as a significant predictor for treatment efficacy after the relief phase, suggesting that treatment efficacy not only dynamically changes over the course of a single treatment, but also that treatment-related evaluations during an early phase of treatment modulate subsequent evaluations during later treatment phases. This may seem trivial at first glance but is in fact intriguing in its putative implication for clinical treatment settings where patients receive the same treatment for longer periods of time, on multiple occasions, and/or in different doses. For the pain phase, on the other hand, a significant model emerged only after exclusion of outliers, with pre-treatment expectations explaining 29% of the variability in efficacy. Together, these results support that interindividual variability in the level of positive treatment expectations arising from positive drug-related information prior to treatment explains variability in perceived treatment efficacy assessed after treatment, which is remarkable herein given overall rather highly positive pre-treatment expectations in this placebo group. Even within such an “optimistic” group, inter-individual variability in the extent of positive expectations contributed to treatment satisfaction, most strongly after pain relief, where more than 50% of the variability in perceived efficacy could be explained in our models, which were robust to outliers. It will be intriguing to learn from much-needed prospective clinical work about the impact of the presumably much greater variability in pre-treatment expectations in clinical patients, ranging from very positive to very negative, and hence including not only positive (placebo) but also negative (nocebo) effects on perceived treatment efficacy.

Interestingly, treatment expectations were unable to explain variability in perceived distension-induced pain intensity during the pain phase. For the pain relief phase, on the other hand, a significant model emerged, with treatment expectations explaining 52% of the variance in perceived distension intensity. While this finding would indicate that pre-treatment expectations shape the experience of visceral stimuli when intensity is distinctly reduced, caution in this interpretation is warranted given that the model was not robust to consideration of outliers.

Based on our findings, we speculate that positive expectancy effects may be facilitated by the experience of pain relief, which would be consistent with recently growing appreciation of reward mechanisms in placebo effects (32). It is also conceivable that the experience of pain relief engages cognitive mechanisms integrating predictions with perceptions (33), which may interact with psychological states and traits relevant to gastrointestinal symptoms (34). The unique perceptual characteristics and emotional properties of aversive visceral signals, especially their diffuse and threatening nature (35–38), call for dedicated mechanistic work in the visceral domain, to clarify if our findings in a small sample of healthy individuals are replicable and generalize to patient populations. Indeed, visceral pain-related expectancy effects are of particular relevance to the treatment of patients with disturbed gut-brain interactions like IBS who commonly experience fluctuating symptoms, and rarely achieve immediate symptom relief with available treatment options. Especially in these patient groups is it likely that treatment expectations dynamically change over time, and are influenced by treatment experiences, including prior treatment successes and failures. At the same time, patients with disorders of gut-brain interactions benefit from psychological treatment approaches (39), which could be further informed by knowledge derived from placebo research to elucidate predictors of treatment satisfaction (40). While our comparatively short experimental paradigm captured the experience of pain relief, we did not model fluctuating symptoms or analyze dynamic changes in pain. Further, we did not have control groups to assess order effects (i.e., herein the pain relief phase was always preceded by the pain phase) or carry-over effects involving learning/experience across or within treatment phases. Indeed, treatment outcome appears to be shaped by expectations arising from prior treatment history. Such “carry-over” or generalization effects have been elegantly shown for nocebo effects in experimental somatic pain (41, 42). While our statistical models for the relief phase did include appropriate covariates (i.e., intensity and/or efficacy of the pain phase), ideally future experimental paradigms would include placebo groups and conditions with and without the experience of pain relief, as well as nocebo groups with and without the experience of pain increase. Clearly, the clinical treatment reality is much more complex and difficult to model in the laboratory in all its facets and intricate interactions. Dedicated translational studies within and beyond the visceral domain are needed to elucidate specific factors, especially the temporal dynamics of changes in positive and negative treatment expectations, symptom experience, and perceived treatment efficacy, as previously suggested (reviewed in Ref. 1).

Our experimental findings in acute visceral pain match observations from clinical trials and longitudinal studies in the broader field of acute (43, 44) and chronic pain (45, 46), which underscore the relevance of pre-treatment expectations for clinical and patient-reported outcomes, including overall treatment efficacy (47). For instance, in a large multicentre, observational study of a multidisciplinary treatment for chronic pain, Cormier et al. (48) demonstrated the impact of treatment expectations on clinical outcomes (e.g., pain intensity, depressive symptoms, pain catastrophizing). Interestingly, this association was mediated by the patients’ global impression of change, pointing to treatment efficacy as multifactorial construct, which might be insufficiently explained by mere pain intensity and the relevance of semi-subjective, patient-reported outcomes. Furthermore, in a meta-analysis Vase et al. (16) demonstrated that approximations of treatment expectations in clinical trials (based on the number of interactions with healthcare professionals and knowledge of an opioid rather than a non-opioid drug as the active comparator) significantly predicted the placebo response in analgesic randomized controlled trials, pointing to the importance of expectations in clinical trials. While patient-reported outcomes are now more frequently implemented in clinical trials (49), standardized assessments of pre-treatment expectations are often still missing (50). This seems even more important considering that–in contrast to this study design–negative expectations toward an active treatment or intervention might even hamper treatment efficacy (42, 51) or lead to adverse events (52). Therefore, future clinical trials should address the relevance of expectations on their outcomes by using standardized tools available [e.g., TEX-Q (53)].

In conclusion, our data from an experimental translational placebo intervention in visceral pain support that pre-treatment expectations shape reported post-treatment medication efficacy. The experience of pain relief may facilitate perceived medication efficacy and by inference treatment satisfaction. Hence, individuals with highly positive expectations may benefit more from a noticeable symptom improvement, and future studies are needed to determine whether the immediate experience of symptoms within a given psychosocial treatment context may dynamically change perceptions about treatment in order to inform and inspire translational studies addressing implications for treatment satisfaction, compliance and adherence in patients with prolonged or spontaneously recurring pain. After all, it has most recently been concluded that “… the patient-physician relationship’s quality is the principal driver of gastroenterology patients’ satisfaction with their care” (54). Enhancing awareness of and knowledge about expectancy effects and their determinants in the context of the gut-brain axis hence holds much promise to further improve the care of the large group of patients with disturbed gut-brain interactions, like IBS, consistent with the vision to maximize positive and minimize negative expectancy effects to the benefit of our patients (55).

## Data Availability Statement

The raw data supporting the conclusions of this article will be made available by the authors, without undue reservation.

## Ethics Statement

The studies involving human participants were reviewed and approved by Essen University Hospital Ethics Committee. The patients/participants provided their written informed consent to participate in this study.

## Author Contributions

NT, LR, and AI acquired data. SE, SB, and NT designed the study and acquired funding. NT, SB, AI, and JK-B analyzed the data. SB, JK-B, and SE wrote the first draft of the manuscript. All authors contributed to the interpretation of the data, revised the manuscript for critical content, and approved the final version of the manuscript.

## Conflict of Interest

The authors declare that the research was conducted in the absence of any commercial or financial relationships that could be construed as a potential conflict of interest.

## Publisher’s Note

All claims expressed in this article are solely those of the authors and do not necessarily represent those of their affiliated organizations, or those of the publisher, the editors and the reviewers. Any product that may be evaluated in this article, or claim that may be made by its manufacturer, is not guaranteed or endorsed by the publisher.

## Footnotes

1
http://www.rstudio.com/
