# Supplementary material for: Positive Treatment Expectations Shape Perceived Medication Efficacy in a Translational Placebo Paradigm for the Gut-Brain Axis
Source: Front Psychiatry. 2022 Mar 24;13:824468. doi: 10.3389/fpsyt.2022.824468 (PMC8987023; doi:10.3389/fpsyt.2022.824468)
Supplement: Supplementary file 1 [file Data_Sheet_1.pdf]

Supplemental Materials

Positive treatment expectations shape perceived medication efficacy in a translational placebo paradigm for the gut-brain axis

Sven Benson, Nina Theysohn, Julian Kleine-Borgmann, Laura Rebernik, Adriane Icenhour, Sigrid Elsenbruch

Table S1: Objective and subjective differences between experimental phases (manipulation check)

	**Objective Distension Pressure** (mmHg)		**Subjective Distension Perception** (VAS)		
	**Pain Phase**	**Relief Phase**	**Pain Phase**	**Relief Phase**	**P***
**Placebo**	33.4 ± 10.4	14.9 ± 3.8	55.3 ± 19.3	17.7 ± 18.3	<.001
**Reference**	33.5 ± 7.2	14.2 ± 3.0	61.8 ± 18.0	16.8 ± 14.5	<.001

Data are shown as mean ± standard deviation, unless otherwise indicated. Objective distension pressures were selected based on individual pain and perception thresholds. For subjective distension perception, repeated measure ANOVA with the factors time (pain, relief) and group (placebo, reference) indicated a significant time effect ($F_{(1,58)}$=193.3, p<0.001), but no significant group ($F_{(1,58)}$=0.5, p=0.49) or interaction effects ($F_{(1,58)}$=1.5, p=0.22). *P results of posthoc computed t-tests.

Table S2: Augmented versus limited medication reminder communication in placebo group

	**Augmented** (N=20)	**Limited** (N=20)	**P**
Medication efficacy after pain phase	62.4 ± 22.1	66.6 ± 12.7	0.47
Medication efficacy after relief phase	73.7 ± 15.2	69.5 ± 21.1	0.47
Perceived intensity during pain phase	57.9 ± 21.4	52.6 ± 17.1	0.39
Perceived intensity during relief	13.4 ± 14.4	22.0 ± 21.1	0.14
phase Tension after pain phase	30.8 ± 35.2	41.8 ± 31.9	0.23
Tension after relief phase	44.6 ± 26.4	37.7 ± 30.1	0.45

Visual analogue scale ratings (mm); shown as mean ± standard deviation. No significant group differences were observed between subgroups (P values indicate results of t-tests).

Table S3: Predictors of treatment efficacy after pain and pain relief phases after exclusion of outliers (Results of Generalized Linear Models, GLM)

**Treatment efficacy after pain phase (R^2 = 0.29)**							
Predictors	*Estimates*	*Std. Beta*	*CI*	*Std. CI*	*t*	*p*	*df*
(Intercept)	50.95	0.00	13.49 –	-0.30 – 0.30	2.66	**0.012**	30
Pre-treatment expectation (VAS)	0.58	0.41	88.45 0.09 – 1.07	0.06 – 0.75	2.31	**0.028**	30
Perceived intensity for pain phase (VAS)	-0.03	-0.04	-0.30 – 0.24	-0.40 – 0.32	-0.23	0.82	30
Stimulus intensity for pain phase (mmHg)	-0.45	-0.31	-1.00 – 0.09	-0.69 – 0.06	-1.64	0.11	30
Tension after pain phase (VAS)	-0.01	-0.02	-0.20 – 0.18	-0.38 – 0.35	-0.08	0.93	30
Duration of medication reminder communication (minutes)	-0.94	-0.19	-2.62 – 0.74	-0.53 – 0.15	-1.09	0.28	30
**Treatment efficacy after pain relief phase (R^2 = 0.62)**							
Predictors	*Estimates*	*Std. Beta*	*CI*	*Std. CI*	*t*	*p*	*df*
(Intercept)	10.05	0.0	-26.06 – 46.16	-0.23 – 0.23	0.55	0.59	27
Pre-treatment expectation (VAS)	0.43	0.29	0.02 – 0.83	0.01 – 0.58	2.06	**0.050**	27
Treatment efficacy rating for pain phase (VAS)	0.60	0.56	0.30 – 0.90	0.28 – 0.85	3.91	**0.001**	27
Perceived intensity for relief phase (VAS)	-0.02	-0.02	-0.29 – 0.25	-0.32 – 0.29	-0.13	0.90	27
Stimulus intensity for relief phase (mmHg)	-0.02	-0.01	-0.44 – 0.40	-0.31 – 0.28	-0.09	0.93	27
Tension after relief phase (VAS)	-0.12	-0.23	-0.29 – 0.05	-0.55 – 0.09	-1.39	0.18	27
Duration of medication reminder communication (minutes)	-0.11	-0.02	-1.56 – 1.33	-0.31 – 0.27	-0.15	0.88	27

Separate generalized linear models (GLMs) with pre-treatment expectation as exploratory and treatment efficacy ratings as response variables were calculated for the pain and pain relief phases, respectively, in positively-instructed volunteers (placebo group). Outliers for VAS treatment expectation and VAS treatment efficacy ratings (defined as values 2 SD below or above mean) were excluded. For a visualization, see Fig. S1.

CI = confidence interval; df = degree of freedom; Std. = Standardized; t = t value; VAS = visual analogue scale

Table S4: Predictors of Pain intensity during pain and relief phases after exclusion of Outliers (Results of Generalized Linear Models, GLM)

**Subjective pain intensity during pain phase (R^2 = 0.29)**							
Predictors	*Estimates*	*Std. Beta*	*CI*	*Std. CI*	*t*	*p*	*df*
(Intercept)	26.84	0.00	-27.03 – 80.71	-0.30 – 0.30	0.98	0.34	30
Pre-treatment expectation (VAS)	-0.12	-0.06	-0.82 – 0.58	-0.44 – 0.31	-0.33	0.74	30
Treatment efficacy rating for pain phase (VAS)	-0.06	-0.04	-0.52 – 0.41	-0.40 – 0.32	-0.23	0.82	30
Stimulus intensity for pain phase (mmHg)	0.94	0.50	0.28 – 1.61	0.15 – 0.85	2.80	**0.009**	30
Tension after pain phase (VAS)	0.29	0.41	0.06 – 0.51	0.08 – 0.74	2.46	**0.020**	30
Duration of medication reminder communication (minutes)	-0.05	-0.01	-2.30 – 2.20	-0.35 – 0.34	-0.05	0.96	30
**Subjective pain intensity during relief phase (R^2 = 0.50)**							
Predictors	*Estimates*	*Std. Beta*	*CI*	*Std. CI*	*t*	*p*	*df*
(Intercept)	31.14	0 00	-13.57 – 75.86	-0.25 – 0.25	1.37	0.18	29
Pre-treatment expectation (VAS)	0.12	-0.07	-0.67 – 0.43	-0.40 – 0.26	-0.41	0.68	29
Treatment efficacy rating for relief phase (VAS)	0.08	0.07	-0.29 – 0.45	-0.25 – 0.40	0.43	0.67	29
Perceived intensity for pain phase (VAS)	0.32	0.36	0.02 – 0.63	0.02 – 0.69	2.06	**0.048**	29
Stimulus intensity for relief phase (mmHg)	-0.77	-0.46	-1.26 – -0.29	-0.76 – -0.17	-3.11	**0.004**	29
Tension after relief phase (VAS)	0.26	0.42	0.04 – 0.48	0.06 – 0.78	2.29	**0.029**	29
Duration of medication reminder communication (minutes)	-1.65	-0.28	-3.41 – 0.10	-0.58 – 0.02	-1.84	0.075	29

Separate generalized linear models (GLMs) with pre-treatment expectation as exploratory and perceived intensity ratings as response variables were calculated for the pain and pain relief phases, respectively, in positively-instructed volunteers (placebo group). Outliers for VAS treatment expectation and VAS treatment efficacy ratings (defined as values 2 SD below or above mean) were excluded. For a visualization, see Fig. S2.
CI = confidence interval; df = degree of freedom; Std. = Standardized; t = t value; VAS = visual analogue scale

Figure S1

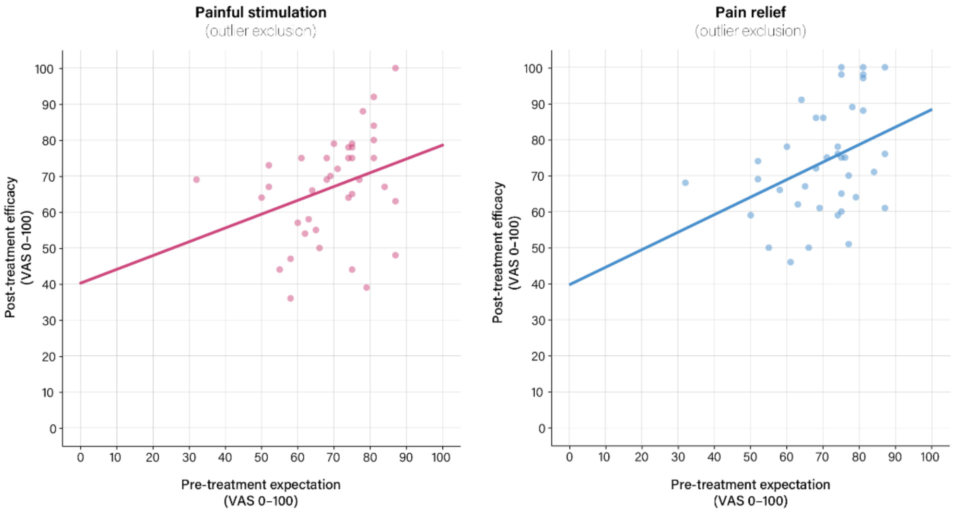

Figure S1: Associations between pre-treatment expectation and perceived treatment efficacy based on general linear models (GLM) calculated using treatment efficacy as exploratory and treatment expectation ratings as response variables after exclusion of outliers. (A) After exclusion of outliers, pre-treatment expectation was significantly associated with treatment efficacy ratings for the pain phase ($b = 0.41$, $t = 2.31$, $p = 0.028$). For the relief phase, the GLM model remained unchanged, i.e., pre-treatment expectations were associated with perceived treatment efficacy ($b = 0.29$, $t = 2.06$, $p = 0.050$). For details, see Table S3.

Figure S2

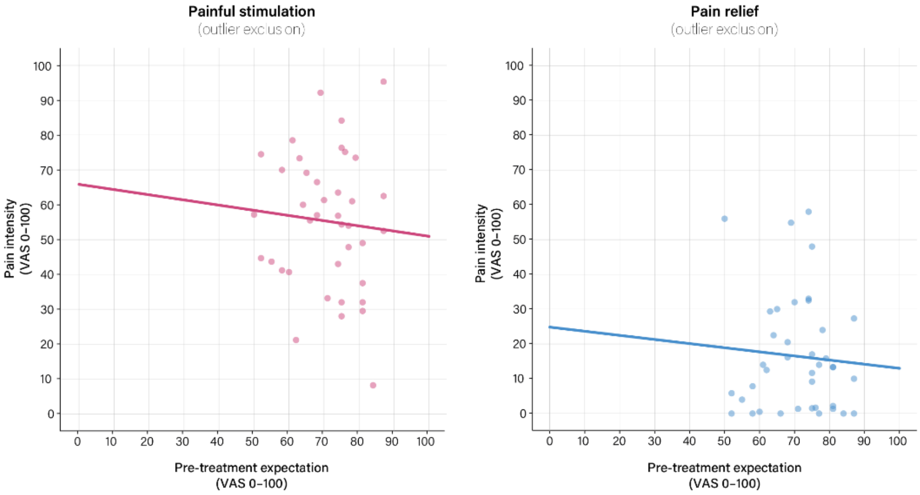

Figure S2: Associations between pre-treatment expectation and perceived distension intensity based on general linear models (GLM) calculated using perceived intensity as exploratory and treatment expectation ratings as response variables after exclusion of outliers. (A) For the pain phase, no significant association between pre-treatment expectation and perceived distension intensity was observed. (B) For the relief phase, the association between pre-treatment expectation and perceived distension intensity was no longer significant after exclusion of outliers. For details, see Table S4.
